# Quantitative susceptibility mapping at 7 T in COVID-19: brainstem effects and outcome associations

**DOI:** 10.1093/brain/awae215

**Published:** 2024-10-07

**Authors:** Catarina Rua, Betty Raman, Christopher T. Rodgers, Virginia F. J. Newcombe, Anne Manktelow, Doris A. Chatfield, Stephen J. Sawcer, Joanne G. Outtrim, Victoria C. Lupson, Emmanuel A. Stamatakis, Guy B. Williams, William T. Clarke, Lin Qiu, Martyn Ezra, Rory McDonald, Stuart Clare, Mark Cassar, Stefan Neubauer, Karen D. Ersche, Edward T. Bullmore, David K. Menon, Kyle Pattinson, James B. Rowe, on behalf of the Cambridge NeuroCOVID group, the CITIID-NIHR COVID-19 BioResource Collaboration, the Oxford CMORE-NEURO group

**Affiliations:** 1 Wolfson Brain Imaging Centre, University of Cambridge, Cambridge CB2 0QQ, UK; 2 University of Cambridge Centre for Parkinson-plus, University of Cambridge, Cambridge CB2 0QQ, UK; 3 Invicro, Invicro London, Burlington Danes Building, Imperial College London, London W12 0NN, UK; 4 Department of Clinical Neurosciences, University of Cambridge, Cambridge CB2 0QQ, UK; 5 Division of Cardiovascular Medicine, Radcliffe Department of Medicine and Oxford University Hospitals NHS Foundation Trust, University of Oxford, Oxford OX3 9DU, UK; 6 Division of Anaesthesia, University of Cambridge, Cambridge CB2 0QQ, UK; 7 Wellcome Centre for Integrative Neuroimaging, FMRIB, Nuffield Department of Clinical Neurosciences, University of Oxford, Oxford OX3 9DA, UK; 8 Department of Psychiatry, University of Cambridge, Cambridge CB2 0SZ, UK; 9 Department of Addictive Behaviour and Addiction Medicine, Central Institute of Mental Health, University of Heidelberg, Heidelberg 69115, Germany; 10 Medical Research Council Cognition and Brain Sciences Unit, Cambridge CB2 7EF, UK; 11 Cambridge NeuroCOVID Group, University of Cambridge, Cambridge Biomedical Campus, Cambridge CB2 0QQ, UK; 12 CITIID-NIHR COVID-19 BioResource Collaboration, University of Cambridge, Cambridge CB2 0QQ, UK

**Keywords:** coronavirus disease of 2019, quantitative susceptibility mapping, 7T MRI, brainstem, inflammation

## Abstract

Post-mortem studies have shown that patients dying from severe acute respiratory syndrome coronavirus (SARS-CoV-2) infection frequently have pathological changes in their CNS, particularly in the brainstem. Many of these changes are proposed to result from para-infectious and/or post-infection immune responses. Clinical symptoms such as fatigue, breathlessness, and chest pain are frequently reported in post-hospitalized coronavirus disease 2019 (COVID-19) patients. We propose that these symptoms are in part due to damage to key neuromodulatory brainstem nuclei. While brainstem involvement has been demonstrated in the acute phase of the illness, the evidence of long-term brainstem change on MRI is inconclusive. We therefore used ultra-high field (7 T) quantitative susceptibility mapping (QSM) to test the hypothesis that brainstem abnormalities persist in post-COVID patients and that these are associated with persistence of key symptoms.

We used 7 T QSM data from 30 patients, scanned 93–548 days after hospital admission for COVID-19 and compared them to 51 age-matched controls without prior history of COVID-19 infection. We correlated the patients’ QSM signals with disease severity (duration of hospital admission and COVID-19 severity scale), inflammatory response during the acute illness (C-reactive protein, D-dimer and platelet levels), functional recovery (modified Rankin scale), depression (Patient Health Questionnaire-9) and anxiety (Generalized Anxiety Disorder-7).

In COVID-19 survivors, the MR susceptibility increased in the medulla, pons and midbrain regions of the brainstem. Specifically, there was increased susceptibility in the inferior medullary reticular formation and the raphe pallidus and obscurus. In these regions, patients with higher tissue susceptibility had worse acute disease severity, higher acute inflammatory markers, and significantly worse functional recovery.

This study contributes to understanding the long-term effects of COVID-19 and recovery. Using non-invasive ultra-high field 7 T MRI, we show evidence of brainstem pathophysiological changes associated with inflammatory processes in post-hospitalized COVID-19 survivors.

## Introduction

Neuroradiological changes have been described in severely affected hospitalized patients with severe acute respiratory syndrome coronavirus (SARS-CoV-2) causing coronavirus disease 2019 (COVID-19). The most common acute findings are cerebral microhemorrhages, encephalopathy and white matter hyperintensities.^1–9^ Brainstem involvement in COVID-19 has also been reported in autopsy studies,^10,11^ which show tissue neurodegeneration and inflammatory responses. These abnormalities are reflected by MRI-visible changes in the brainstem in the acute phase of the illness.^8^ Indeed, such brainstem abnormalities have been proposed^12,13^ as a mechanism for post-acute COVID syndrome 2 (PACS), which may be related to ‘long-COVID’.^14^ This syndrome includes enduring somatic symptoms (such as fatigue and breathlessness, often in the absence of objectively demonstrable cardiorespiratory abnormalities), cognitive deficits (sometimes referred to as ‘brain fog’) and mental health problems (such as anxiety, depression and post-traumatic stress disorder). However, conventional 3 T MRI has not shown consistent brainstem abnormalities at follow-up. More advanced MRI techniques such as quantitative susceptibility mapping (QSM) have potential to identify more subtle abnormalities, which could reveal neuroanatomical changes in the brainstem after COVID infection.

*In vivo* QSM is a post-processing technique applied to T_2_*-weighted gradient-echo images. Constituents of tissue can contribute a negative (or ‘diamagnetic’) susceptibility (e.g. soft tissue, calcium, myelin) or a positive (or ‘paramagnetic’) susceptibility (e.g. iron, aluminium, copper). QSM is effective to detect cerebral microbleeds,^15^ increases in iron deposition in the basal ganglia and midbrain with age and in disease,^16–18^ to differentiate calcified from haemorrhagic lesions^19^ and to detect chronic inflammation in multiple sclerosis.^20^ Furthermore, high-resolution, ultra-high field (≥7 T) QSM has improved susceptibility contrast in cortical and subcortical tissues,^21^ which provide greater sensitivity to detect microstructural alterations.

By capitalizing on a preliminary analysis showing abnormal brainstem QSM in post-hospitalized patients with COVID-19,^22^ we proceeded to investigate QSM abnormalities in the brainstem, according to subregions [midbrain, pons, medulla and superior cerebellar peduncle (SCP)] defined *a priori* as regions of interest (ROIs). Furthermore, to increase regional specificity, we performed the group analysis using a voxel-by-voxel approach to localize specific clusters in the brainstem showing atrophy. Finally, we tested whether susceptibility in the brainstem correlates with clinical measures of disease severity, laboratory measures of inflammation, and measures of recovery with similar regional-wise and voxel-wise analysis.

## Materials and methods

### Participants

We recruited people who were hospitalized with COVID-19 and subsequently discharged as ‘post-hospitalized patients’ (COVID group; *n* = 31, 18 males, age 57 ± 12 years) for scanning by 7 T MRI at two sites: (i) site-1 at the Wolfson Brain Imaging Centre (WBIC, Cambridge, UK); and (ii) site-2 at the Wellcome Centre for Integrative Neuroimaging (WIN, Oxford, UK) (Supplementary material section 1). Inclusion criteria were: (i) evidence of COVID-19 infection confirmed by SARS-CoV-2 PCR of respiratory samples (nasal or throat swab); (ii) no specific pre-COVID history of neurological or psychiatric disorders; and (iii) no contradictions to 7 T MRI.

COVID-19 severity was determined during hospital admission using the World Health Organization (WHO) ordinal scale for clinical improvement.^23^ Peak C-reactive protein (CRP) and D-dimer levels, and lowest platelet levels during hospital stay were recorded. At the time of the follow-up clinic (time between clinic and imaging was 50 ± 21 days for site-1 and 115 ± 34 for site-2), functional recovery was assessed using the modified Rankin scale (mRS, at site-1 only), and mental health was assessed using two sets of questionnaires for anxiety and depression, respectively, the Generalized Anxiety Disorder-7 (GAD-7) and the Patient Health Questionnaire-9 (PHQ-9).

Healthy controls (HC) were scanned by 7 T MRI in site-1 (HC group, *n* = 51, 34 males, age 53 ± 15 years). These came from three subgroups: people scanned before December 2019, i.e. before possible exposure to COVID-19 (‘HC1 and HC2 subgroups’, *n* = 18 and *n* = 24, respectively); and people scanned during the pandemic before April 2021 who were asymptomatic with no history of positive SARS-CoV-2 PCR (‘HC3 subgroup’, *n* = 9) (Supplementary material section 1).

The study was approved by the following ethics committees: Cambridgeshire Research Ethics Committee HBREC.2016.13.am3, East of England Research Ethics Committee 17/EE/0025, Norfolk Research Ethics Committee EE/0395 and Northwest Preston Research Ethics Committee 20/NW/0235. All participants provided informed consent in accordance with the Declaration of Helsinki.

### MRI acquisition

All participants had 7 T MRI using a 32-channel head coil (Nova Medical). Site-1 used their 7 T Terra scanner (Siemens) and site-2 used their Magnetom 7 T (Siemens). Following previous published results on the reproducibility across these two scanners for QSM,^24^ both sites acquired 3D T_2_*-weighted multi-echo gradient-echo with 0.7 mm isotropic voxels, 4.68 ms echo time (TE1), 27 ms repetition time (TR), six echoes, 3.24 ms echo spacing, 15° nominal flip angle, 430 Hz/pixel bandwidth, 2 × 2 acceleration-factor, over a 224 × 196 × 157 mm^3^ field of view. Magnetization prepared 2 rapid gradient echo (MP2RAGE) T_1_-weighted scans were acquired for anatomical localization and registration. For the COVID and HC2 and HC3 groups this used the UK7T harmonized protocol^25^: 0.7 mm isotropic voxels, 2.64 ms echo time, 3500 ms TR, 300 Hz/pixel bandwidth, 725/3150 ms inversion time (TI), 5°/2° nominal flip angles and 224 × 224 × 224 matrix. For the HC1 group we used: 0.75 mm isotropic voxels, 1.99 ms TE, 4300 ms TR, 250 Hz/pixel bandwidth, 840/2370 ms TI, 5°/6° nominal flip angles and 240 × 224 × 168 matrix.

### Data processing

Image processing used routines from the advanced normalization tools (ANTs) v2.2.0, FMRIB software library (FSL) v6.0.1, statistical parametric mapping library (SPM12) v7219 and MATLAB R2018b. Per channel data were combined as previously described at 7 T.^26^ Quantitative susceptibility (χ) maps were estimated from the coil-combined T_2_^*^-weighted phase data using the multi-scale dipole inversion algorithm in QSMbox,^27^ as previously described.^24^ The reference region used in the QSM analysis was the whole brain χ value. T_1_-weighted structural images were computed from the raw images as previously described^24^ for the HC2, HC3 and COVID groups, and using the vendor supplied method for the HC1 group. All T_1_-weighted scans were then bias-field corrected with ANTs, segmented with SPM12 and skull-stripped. We transformed the per-subject susceptibility maps into the 0.5 mm isotropic ICBM 2009b standardized space for statistical analysis as described in Supplementary material section 2.

### Region of interest analysis

Brainstem ROIs were defined using the ‘-brainstem-structures’ tool^28^ in FreeSurfer (v6.0.0) on the ICBM T_1_-weighted image to extract ROIs for brainstem and four subregions: the midbrain, pons, medulla and superior cerebellar peduncle (SCP). Mean susceptibility was extracted per ROI and used for further analysis.

QSM data from both sites used matched protocols developed during the UK7T harmonization project.^24,25^ Nevertheless, we tested for possible between-site effects by fitting a linear model to susceptibility at each ROI adding site as a fixed effect, age and sex as covariates, and subject as a random effect. No significant site effects were detected (Supplementary material section 3), and hence further analyses further analyses treated the COVID data as a single group.

We fitted linear models separately for each ROI, with group (COVID versus HC) as a fixed effect allowing the intercept to vary across participants (random effect). Considering that there may be age-related trends in QSM χ^16,18^ with associated Gender × Age effects,^29^ we added age, gender and their interaction as covariates. We report frequentist hypothesis testing results, with false discovery rate (FDR)-corrected *P*-value < 0.05 for significance, Cohen’s *d*, 95% confidence interval (CI). We also report Bayesian model comparisons in terms of Bayes factor (BF) and posterior probability [*Pr*(post.)], with BF > 3 defined per conventional criteria as evidence in favour of the alternative hypothesis and BF > 20 as very strong evidence.^30^ Conversely, BF < 1/3 and BF < 1/20 re interested as evidence and very strong evidence for the null hypothesis respectively, which cannot be inferred from ‘non-significant’ frequentist tests’ *P*-values.

### Voxel-wise analyses and association with clinical and laboratory outcomes

Because the brainstem and subregions showed strong group differences (see ‘Results’ section), an additional voxel-wise analysis comparing the COVID versus HC groups within the brainstem ROI was undertaken to improve the resolution for spatial distributions of small cluster differences (note the emphasis was localization of clusters in the voxel-wise analysis, not the significance of differences given the non-independence of ROI in the voxel-wise tests). The co-registered susceptibility maps were masked by the brainstem ROI and subjected to general linear models for testing group differences.

The voxel-wise analysis was performed with the ‘Randomise’ function in FSL, setting the number of permutations to 7000, and the threshold free cluster enhancement (TFCE) method for cluster inference. Within these analyses, models included Age, Gender and Age × Gender interaction as covariates. To isolate the most significant clusters, a conservative family-wise error (FWE) corrected *P*-value < 0.01 was used to determine significant voxels, and the function ‘cluster’ in FSL was applied to group significant clusters. We report also FWE-corrected *P*-value < 0.05 results in the Supplementary material, for reference. The centroid and spatial extent of the clusters were evaluated in Montreal Neurological Institute (MNI) space. The brainstem Navigator Atlas (https://brainstemimaginglab.martinos.org/research/) ROIs that overlapped the significant clusters were reported for spatial identification of the cluster location.

From the patient data, we extracted the mean χ from the significant clusters (FWE threshold *P*-value < 0.01) and fitted nine linear models to test their association with clinical and laboratory outcomes (WHO score, period of hospital admission, highest CRP during admission, highest D-dimer during admission, lowest platelets during admission, GAD-7, PHQ-9 and mRS). As some clinical and laboratory measurements were not available for all subjects, we performed the linear mixed effects model by dropping patient data that did not include the measurement of interest (Supplementary material section 4).

Each model also included Age, Gender, Age × Gender interaction, time from hospital admission to scan and cluster number as fixed effects, and subject as a random effect. The Shapiro–Wilk test was used to test normality of the outcome variable, and the model fit was evaluated for multicollinearity, normality distribution of residuals and homoscedasticity with the package *sjPlot* from R. When testing for the effect of period hospital admission, one subject (hospital admission 134 days) was an extreme outlier and was excluded (Supplementary material section 5). Nonetheless, results obtained with retention of the outlier were essentially the same as the main results reported below (Supplementary material section 10). We report both frequentist hypothesis testing results and Bayesian statistics.

## Results

### Demographic and clinical characteristics

Overall, there were no significant differences between the two groups in age, but there were more males than females in the HC group compared with the COVID group (*χ*^2^ = 4.92, *P* = 0.03). One subject from Site 1 presented a very low QSM signal (due to lack of signal in the brainstem; Supplementary material section 6) and was excluded from the analysis. Consequently, data from 51 healthy controls and 30 patients were used for further analysis. The demographic and clinical features of participants used in the analyses are shown in Table 1. In the COVID group, the median time from hospital admission to the MRI scan was 199 days.

### Group differences

**Figure 1:**
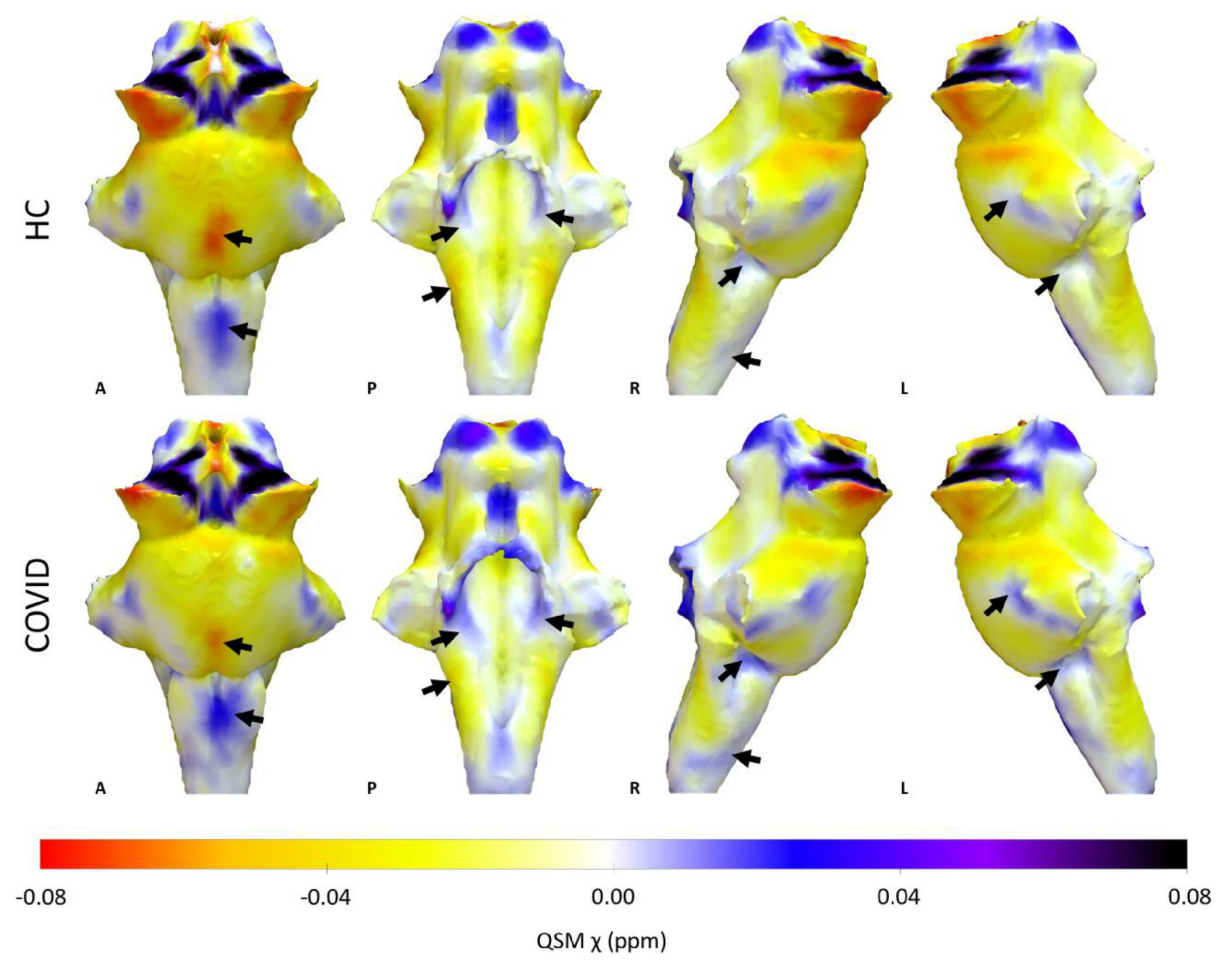
3D projections of the quantitative susceptibility mapping χ maps on the rendered brainstem regions of interest extracted from the FreeSurfer segmentation for the healthy control group and the COVID group. The coronavirus disease 2019 (COVID) group shows increased χ in the brainstem, specifically in the medulla and pons (black arrows). A = anterior; HC = healthy control group; L = left; P = posterior; QSM = quantitative susceptibility mapping; R = right. 3D renderings were generated with Surf Ice.

**Figure 2:**
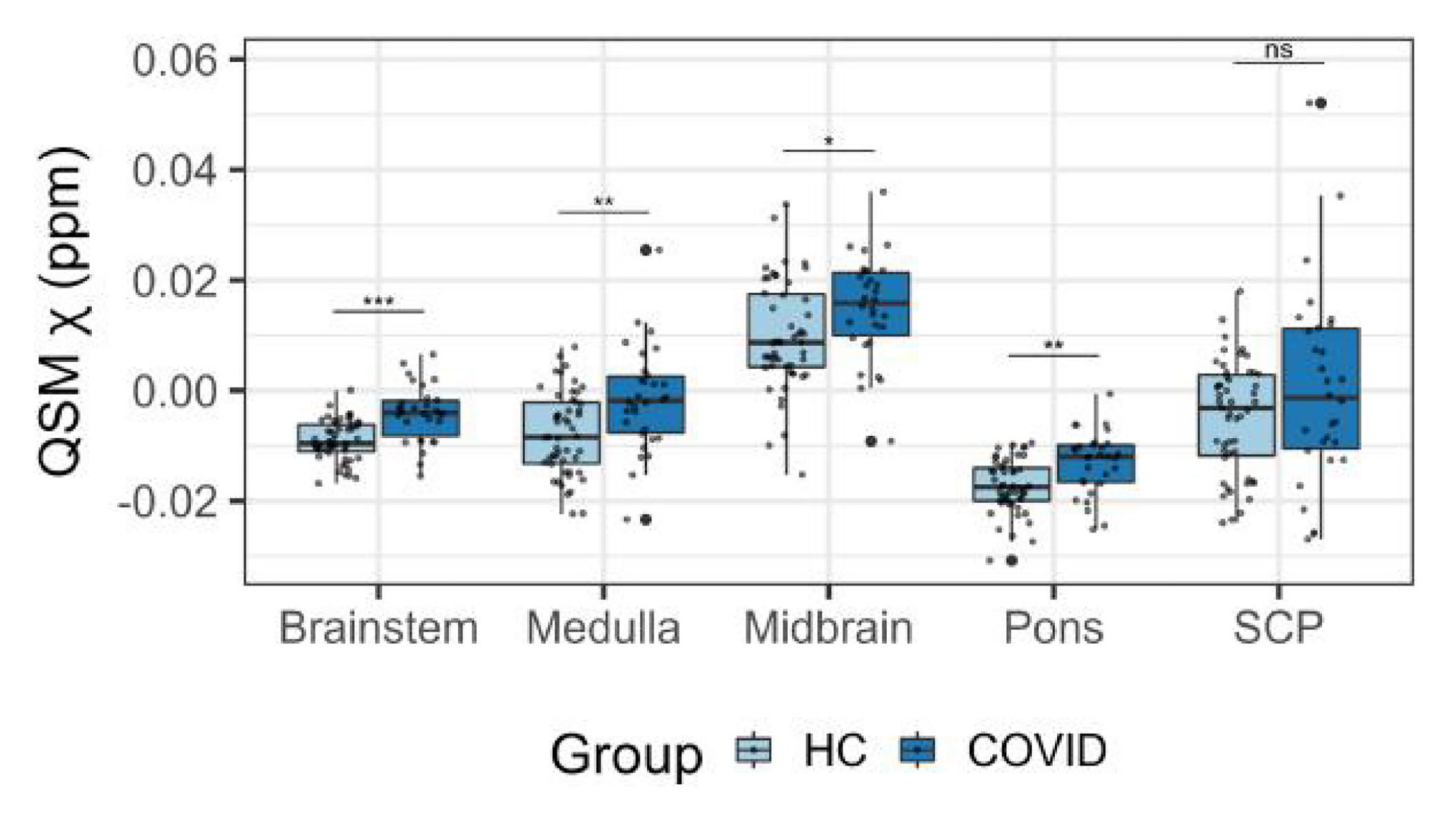
Box plots of differences in the regional average χ between the COVID group and the healthy control group obtained from the brainstem. Group differences assessed with a linear model with age, gender and age by gender interactions added as explanatory variables of no interest. False discovery rate-corrected statistics represented on the box plots. ***P < 0.001, **P < 0.01, *P < 0.05, ns = not significant. COVID = coronavirus disease 2019; HC = healthy control; QSM = quantitative susceptibility mapping; SCP = superior cerebellar peduncle.

The regional mean *χ* increased in the brainstem in the COVID group compared with the HC group [Figs 1 and 2; *P*(FDR) < 0.0001, *d* = 4.96, 95%CI (0.0030, 0.0071), *Pr*(post.) = 1.00, BF = 4688]. This significant increase in *χ* was mainly localized in the pons subregion of the brainstem [Fig. 2; *P*(FDR) = 0.00042, *d* = 4.01, 95%CI (0.0024 0.0071), *Pr*(post.) = 0.99, BF = 108] and in the medulla [Fig. 2; *P*(FDR) = 0.0035, *d* = 3.37, 95%CI (0.0027, 0.010), *Pr*(post.) = 0.96, BF = 23]. The midbrain subregion only showed a weak significant group effect [Fig. 2; *P*(FDR) = 0.032, *d* = 1.99, 95%CI (0.0000042, 0.0091), *Pr*(post.) = 0.69, BF = 2.27]. There was no group difference in the SCP (Fig. 2).

**Figure 3:**
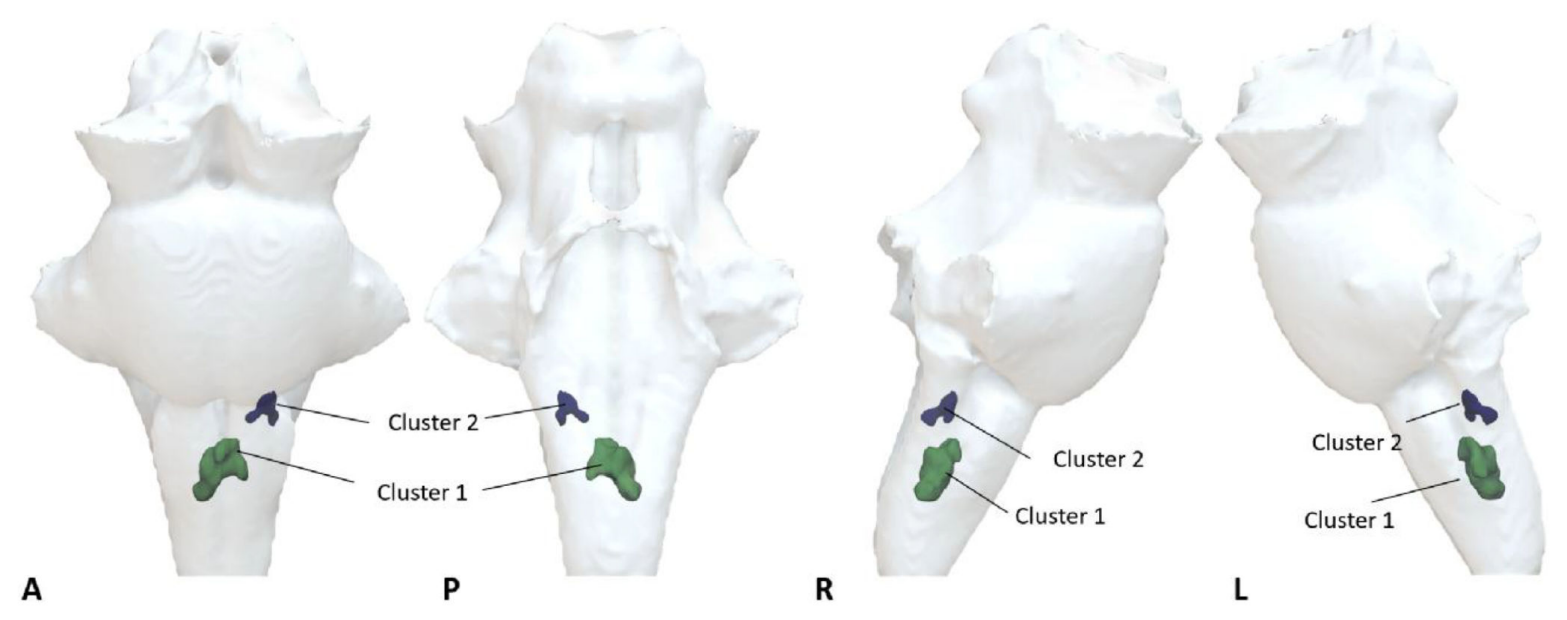
Voxel-wise analysis showing increased quantitative susceptibility mapping χ on the COVID group compared with healthy controls. Significant clusters determined with the ‘Randomise’ function in FSL [threshold free cluster enhancement (TFCE) corrected *P* < 0.01, cluster inference t = 2.5, cluster volume > 1 mm^3^]. 3D projection of the significant clusters on the brainstem region of interest. A = anterior; COVID = coronavirus disease 2019; L = left; P = posterior; R = right. 3D renderings were generated with Surf Ice

Voxel-wise analyses on the brainstem identified two significant clusters located in the medulla region (Fig. 3 and Table 2) with significant increase in *χ* in the COVID group [Cluster 1: *P*(FDR) < 0.0001, *d* = 4.56, 95%CI (0.011, 0.028), *Pr*(post.) = 1.00, BF = 1073; Cluster 2: *P*(FDR) < 0.0001, *d* = 4.86, 95%CI (0.0098, 0.023), *Pr*(post.) = 1.00, BF = 906]. These clusters partially overlap brainstem regions known to be associated with respiratory function and body homeostasis, including the inferior medullary reticular formation nuclei, the raphe obscurus and pallidus. At the less stringent, but still significant, threshold corrected for multiple comparisons (FWE threshold *P* < 0.05), additional clusters were observed in the pons and midbrain subregions of the brainstem (Supplementary material sections 7–9).

### Brainstem pathology and clinical assessments in patients

**Figure 4:**
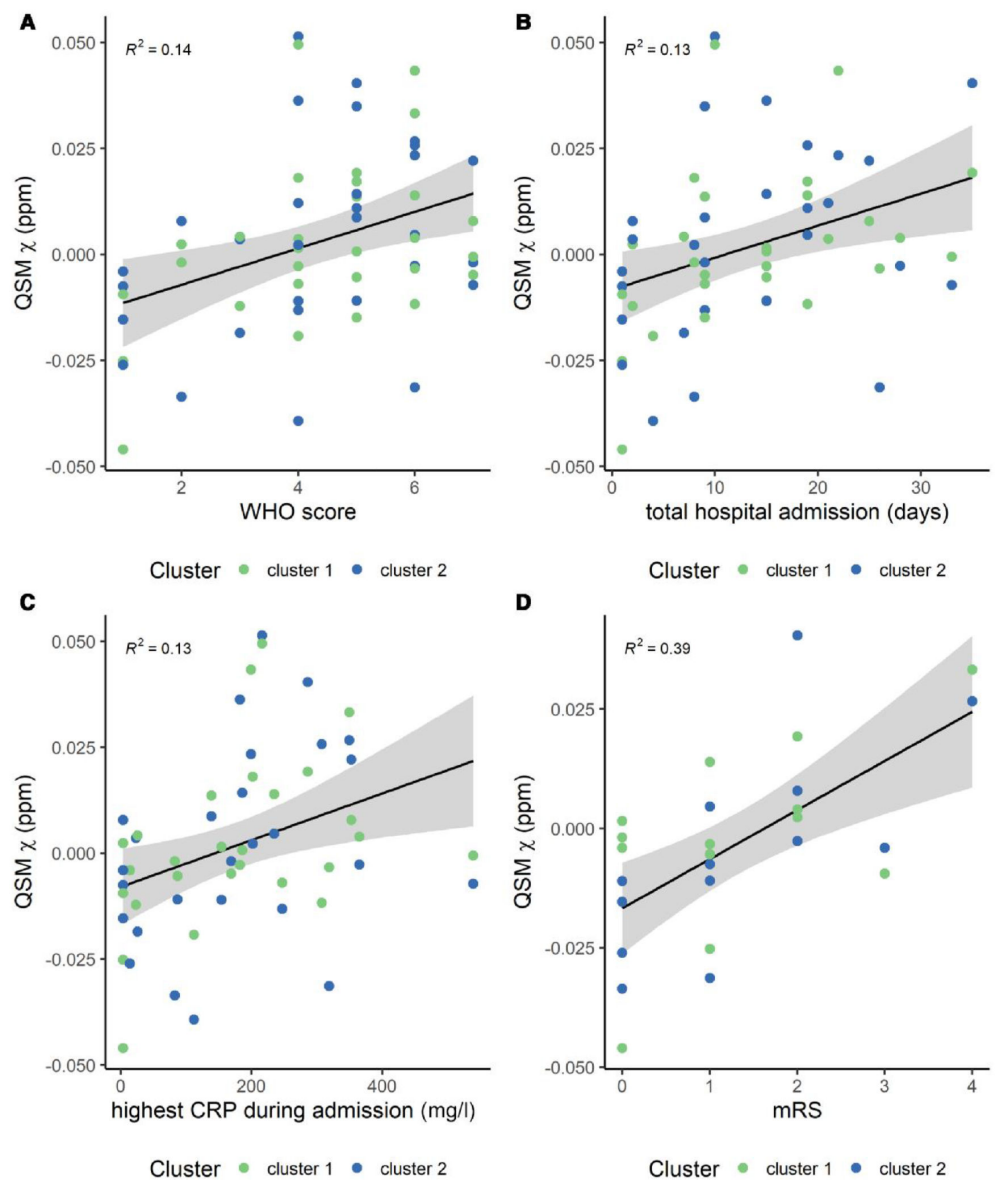
Scatter plots of the average quantitative susceptibility mapping (QSM) χ obtained on the clusters from the voxel-wise group analysis with clinical and laboratory outcomes. (**A**) World Health Organization (WHO) score, (**B**) period of hospital admission, (**C**) highest C-reactive protein (CRP) during admission and (**D**) modified Rankin Score (mRS). The *R*^2^ value is also displayed on each plot. **B** shows data without the outlier (Supplementary material section 5).

The mean *χ* values extracted from the two clusters identified in the medulla portion of the brainstem (FWE threshold *P* < 0.01) were positively associated with the highest CRP detected during admission [*R* = 0.36, *P* = 0.041, *Pr*(post.) = 0.84, BF = 5.1] and mRS [*R* = 0.60, *P* = 0.0046, *Pr*(post.) = 0.94, BF = 16.3]. It was also weakly associated with the WHO severity index [*R* = 0.40, *P* = 0.046, *Pr*(post.) = 0.70, BF = 2.3] and period of hospital admission [*R* = 0.37, *P* = 0.054, *Pr*(post.) = 0.7, BF = 3.1] (Fig. 4 and Supplementary material section 10). There were no significant trends for other laboratory variables and clinical assessments (for highest D-dimer during admission, GAD-7, PHQ-9 and lowest platelets during admission, *R* < 0.21, *P* > 0.15, *Pr*(post.) < 0.47 and BF < 0.88). No significant effect was found between the two clusters (for all variables, *P* > 0.60, *Pr*(post.) < 0.29, BF < 0.40) (Supplementary material section 10). Scatter plots (Fig. 4) display the average *χ* extracted from the two clusters against the significant clinical outcome variables.

## Discussion

This study provides imaging evidence for mid- to long-term microstructural abnormalities in the brainstem following COVID-19 hospitalization. Our key findings were that in COVID-19 survivors, multiple regions of the medulla oblongata, pons and midbrain show magnetic resonance susceptibility abnormalities at a median time of 6.5 months from hospital admission. These differences are consistent with a neuroinflammatory response. The fact that these regions that were affected several weeks after hospitalization are the sites of respiratory pathways suggests that lasting symptoms might be an indirect effect of brainstem inflammatory injury following COVID-19. Note, however, that our study was not designed to test whether there is a direct or indirect mechanisms of injury. These effects are independent of age and gender, and were more pronounced in those who had had more severe initial COVID-19 illness.

Symptoms of fatigue, dyspnea, breathlessness, cough and chest pain are common in the months after COVID-19 infection.^31–36^ Brainstem changes may predispose to, or exacerbate, such symptoms over and above peripheral organ damage. This role in the aetiology of long-term symptoms may arise because the brainstem provides a nexus between sensory and motor inputs, and between the spinal cord and the brain, with nuclei that are responsible for controlling the sleep-wake cycle, respiratory drive, cardiac and vasomotor regulation. We hypothesize that a brainstem insult follows COVID-19 in hospitalized patients, impairing autonomic functions that contribute to persisting clinical symptoms. In part, a similar pattern is observed following post severe traumatic brain injury, with patients reporting fatigue and dizziness but also tachycardia, tachypnoea and hypertension,^37,38^ linked to acute or chronic brainstem dysfunction.^39^

Neuropathological changes in the brainstem in patients with COVID-19 have been detected post-mortem.^11^ In most cases, there is no evidence of direct viral infection of the CNS but rather a neuroinflammatory response to systemic infection. The process of increase in χ in patients recovering from COVID-19 infection is reminiscent of the observed inflammatory response in other neuroinflammatory disorders such as multiple sclerosis.^40,41^ In COVID-19, we hypothesize that an indirect effect of the SARS-CoV-2 virus is to cause similar iron dysregulation via microglia activation. During acute inflammation, macrophage iron levels rise^42^ in concert with increased production of cytokines and reactive oxygen species.^43^ Indeed, an increase of intracellular iron content can itself promote a proinflammatory state.^44^ Increased susceptibility might also reflect a loss of myelin, whether directly or indirectly, as a consequence of neuroinflammation. However, the loss of myelin is typically a slower process than autoimmune neuroinflammation.

Approaches for χ-separation have been proposed that attempt to attribute the individual contribution of paramagnetic iron and diamagnetic myelin susceptibility sources from the frequency shift and transverse relaxation of MRI signals.^45^ In future studies, these methods could be applied to disambiguate the interpretation of the brainstem susceptibility changes observed in post-hospitalized COVID-19 patients.

Our analysis was focused on the brainstem, exploring changes not only its subregions (midbrain, pons, medulla and SCP) but also on a voxel-by-voxel basis to allow increased anatomical resolution. The latter approach highlighted clusters in the inferior medullary reticular formation and in the raphe obscurus and pallidus, with increased tissue susceptibility in the COVID group compared with HCs. The medullar reticular formation contains neurones that are responsible for the central control of the respiratory cycle. Nuclei included in the formation include the dorsal respiratory group and the ventral respiratory group (with inhibitory and pre-motor expiration neurons).^46,47^ In addition, neurons in the raphe pallidus and obscurus have been found to be central chemoreceptors^47^ responsible for the full expression of ventilatory responses to hypercapnia.^48^ We propose that these changes provide evidence of a viral-induced proinflammatory state, which is responsible for impaired function in key brainstem circuits generating and controlling physiological allostasis.

CRP is a non-specific marker of inflammation or infection and has been found elevated in patients with COVID-19 and other acute respiratory syndromes such as the H1N1 influenza virus.^49^ Our results showed that patients with a greater peak inflammatory response during hospital admission (peak CRP) exhibited increased tissue susceptibility (likely associated with increased inflammation) in clusters within the medulla responsible for a regular autonomic respiratory function. In turn, patients with a more favourable functional outcome (mRs 0–2), with shorter hospital stays or lower COVID severity ratings showed decreased susceptibility in the medullary clusters. COVID-19 appears to drive a post-viral, long-lasting, hyperactivation of the immune system within the brainstem, impairing certain autonomic functions. In a similar manner, a portion of SARS and Middle East respiratory syndrome survivors have shown similar long-lasting post-viral illnesses.^50–52^

In the brain, as first described by Raman *et al*.^53^ and later by Griffanti *et al*.,^54^ susceptibility related changes in COVID-19 patients were found in the thalamus in terms of T_2_* but not χ, which was attributed to differences in tissue compartmentalization. In addition, Griffanti *et al*.^54^ found differences in χ in the right hippocampus. The authors argue that this could be related to higher iron accumulation related to virus infection but could also be a partial volume issue of the MRI acquisition. An earlier analysis of a subset of our dataset^22^ did not show any QSM χ changes in these regions at 7 T (which would be expected to enhance tissue susceptibility differences). Analysis of our full dataset consistently showed no group effects in the thalamus, hippocampus (Supplementary material section 11) or any other high-iron subcortical brain structures. However, our patients were scanned on average 219 days after hospital admission, which is over 3.5-times longer than the timing of scans in these prior studies.^53,54^ Many brain changes normalize at 6-month follow-up imaging,^55,56^ and these differences in scan timing could contribute to the difference in the results observed with our dataset.

Many studies have demonstrated that ultra-high field phase imaging improves contrast-to-noise ratio of cortical regions or iron-rich regions such as the globus pallidus or substantia nigra that have been used to assess changes in pathology.^21,57,58^ In this study, we were able to highlight the importance of ultra-high field imaging to detect changes in the brainstem that were not previously reported (Supplementary material section 12). At 7 T, QSM was able to detect negative and diffuse susceptibility values which were, on average across all HCs, −0.0091 ± 0.0037 ppm. In contrast, COVID-19 patients exhibited a susceptibility value of −0.0042 ± 0.0052 ppm. Although, on average, the change in absolute χ is only approximately 5 parts per billion, we propose this significant result to be a biologically meaningful increase of susceptibility in the brainstem of the COVID-19 patients. Normative values for this brain region are lacking in the literature, and we interpret the results to reflect high signal-to-noise ratio, with sufficient precision for the neuroimaging arising from our *n* > 50 control group.

The study has several limitations. The sample size of patients was relatively small and heterogeneous. Recruitment of patients was challenging due to the contemporary safety concerns and lockdowns before the widespread availability of vaccines. This study was a multi-centre effort. Our imaging results were indicative of negligible site effects for QSM providing increasing confidence on the applicability of T_2_* imaging for the CNS in multi-centre trials. We also acknowledge the gender imbalance of our normative dataset. The cohort was partly formed of data from a number of clinical studies acquired prior to the COVID-19 outbreak which contained a different gender balance. For this study, we extracted a sample of HCs, selecting control cohorts where we were confident that the subjects had not experienced clinical or subclinical SARS-CoV-2 infection. In addition, all our group analyses were controlled for gender and age effects, and their interaction. For the voxel-wise assessment, we used a conservative FWE threshold *P*-value of 0.01 for cluster inference and found two small clusters in the medulla region of the brainstem. This allowed us to isolate the most prominent peak locations that showed changes in our patient group compared with controls. At a lower threshold, other regions in the pons and midbrain showed increases in tissue susceptibility for the COVID patients, overlapping with the inferior olivary nucleus, the pontis oralis and caudalis, the ventral tegmental area, the periaqueductal gray and others (Supplementary material section 7). Future work utilizing brainstem MR susceptibility as a proxy of brain inflammation, together with further clinical indexes of sleep-wake cycle and cardiovascular and respiratory control metrics, might allow further understanding about which brainstem regions become impaired and to which extent. We also acknowledge that these scans were taken on a single time point after hospitalization (on average 6 months after hospitalization). Prospective follow-up studies would be helpful to understand the long-term sequelae of COVID-19 hospitalization.

In conclusion, we show that the brainstem is a site of vulnerability to long-term effects of COVID-19, with persistent changes evident in the months after hospitalization. These changes were more evident in patients with longer hospital stays, higher COVID severity, more prominent inflammatory responses and worse functional outcomes. Ultra-high field 7 T QSM was sensitive to these pathological changes in the brainstem, which could not be detected at standard clinical field strengths. This approach can provide a valuable tool to better probe the brain for the long-term effects of COVID-19 and other potential SARS-CoV diseases, in order to inform acute and long-term therapeutic strategies to aid recovery.

## Supplementary Material

Supplementary Material

## Figures and Tables

**Table 1:** Demographics, clinical and scan data of the subjects used in the analysis of this study

	HC	COVID
*n*	51	30
Age, years	53 ± 15	57 ± 12 (*t*-test, *P* = 0.32)^a^
Gender	34 M, 17 F	18 M, 13 F (χ^2^ = 6.87, *P* = 0.032)^a^
Period of hospital admission, days	–	17 ± 24
Time from admission to 7 T MRI scan, days	–	219 ± 84
Time from follow-up clinic to 7 T MRI scan, days	–	79 ± 42
Highest CRP during admission, mg/l	–	178 ± 139
Highest D-dimer during admission, ng/ml	–	4883 ± 13 188
Lowest platelets during admission, 10^9^/l	–	199 ± 50
WHO severity scale, range: 0–10	–	4.3 ± 1.9
GAD-7, range: 0–21	–	4.7 ± 5.5 (range: 0–20)
PHQ-9, range: 0–27	–	6.7 ± 5.1 (range: 0–16)
Modified Rankin Score	–	Median = 1.0, range: 0–4; IQR = 2.0

Within-group mean ± standard deviation values reported when appropriate. Disease severity, blood serum values and clinical assessments are reported for the coronavirus disease 2019 (COVID-19) group. CRP = C-reactive protein; F = females; HC = healthy controls; IQR = interquartile range; GAD-7 = Generalized Anxiety Disorder assessment; M = males; PHQ-9 = Patient Health Questionnaire-9; WHO = World Health Organization.

a Tests comparing the HC and COVID groups.

**Table 2:** Cluster characteristics and statistics

	Cluster 1^a^	Cluster 2^a^
Volume of cluster, mm^3^	96.75	21.13
Maximum *t*-statistic in cluster	5.83	4.79
COG X	179	192
COG Y	167	169
COG Z	28	43.5
Location	Medulla	Medulla
Brainstem Navigator ROIs overlapping cluster	Inferior medullary reticular formation (left and right), raphe obscurus, raphe pallidus	Inferior medullary reticular formation (left)

Volume, maximum *t*-statistic, centre of gravity (COG), location in the brainstem and overlapping Brainstem Navigator regions of interest (ROIs) from the significant clusters determined with the ‘Randomise’ function in FSL [threshold free cluster enhancement (TFCE) corrected *P* < 0.01, cluster inference *t* = 2.5, cluster volume > 1 mm^3^].

a Clusters shown in Fig. 3.

## Data Availability

We can provide average QSM χ extracted values from the brainstem and subregions upon reasonable request.
