# Correction to: Quantitative susceptibility mapping at 7 T in COVID-19: brainstem effects and outcome associations

**DOI:** 10.1093/brain/awae332

**Published:** 2024-10-28

Catarina Rua, Betty Raman, Christopher T. Rodgers, Virginia F. J. Newcombe, Anne Manktelow, Doris A. Chatfield, Stephen J. Sawcer, Joanne G. Outtrim, Victoria C. Lupson, Emmanuel A. Stamatakis, Guy B. Williams, William T. Clarke, Lin Qiu, Martyn Ezra, Rory McDonald, Stuart Clare, Mark Cassar, Stefan Neubauer, Karen D. Ersche, Edward T. Bullmore, David K. Menon, Kyle Pattinson, James B. Rowe, on behalf of the Cambridge NeuroCOVID group, the CITIID-NIHR COVID-19 BioResource Collaboration and the Oxford CMORE-NEURO group. Quantitative susceptibility mapping at 7 T in COVID-19: brainstem effects and outcome associations. *Brain*. 2024;147:4121–4130. https://doi.org/10.1093/brain/awae215

The authors apologize for errors in the ‘Acknowledgements’ and ‘Funding’ sections of the originally published paper.

The ‘Acknowledgements’ section should read: ‘We thank our research participants for their invaluable dedication and inspiration. We thank NIHR BioResource volunteers for their participation, and gratefully acknowledge NIHR BioResource centres, NHS Trusts and staff for their contribution. We thank the National Institute for Health and Care Research, NHS Blood and Transplant, and Health Data Research UK as part of the Digital Innovation Hub Programme. We thank the radiography, ethics and admin support teams at both sites for their involvement in the delivery of this project, especially developing new procedures for safe research during the COVID pandemic.’ instead of: ‘We thank our research participants for their invaluable dedication and inspiration. We thank the radiography, ethics and admin support teams at both sites for their involvement in the delivery of this project, especially developing new procedures for safe research during the COVID pandemic.’

The first two sentences of ‘Funding’ section are emended to read: ‘This work was supported by the NIHR Cambridge Biomedical Research Centre [NIHR203312; BRC-1215-20014], by the NIHR BioResource [RG94028, RG85445], by the NIHR Oxford Biomedical Research Centre [BRC-1215-20008], and by the University of Oxford Medical Sciences Division COVID-19 Research Response Fund [0009118]. The views expressed are those of the authors and not necessarily those of the NHS, the NIHR or the Department of Health and Social Care.’ instead of: ‘This work was supported by the NIHR Cambridge Biomedical Research Centre [NIHR203312; BRC-1215-20014], by the NIHR Oxford Biomedical Research Centre [BRC-1215-20008], and by the University of Oxford Medical Sciences Division COVID-19 Research Response Fund [0009118]. The views expressed are those of the authors and not necessarily those of the NIHR or the Department of Health and Social Care.’

Emendations have been made to the article online.

