# Supplementary material for: Quantitative susceptibility mapping at 7 T in COVID-19: brainstem effects and outcome associations
Source: Brain. 2024 Oct 7;147(12):4121–30. doi: 10.1093/brain/awae215 (PMC7616766; doi:10.1093/brain/awae215)
Supplement: awae215_Supplementary_Data [file awae215_supplementary_data.pdf]

Supplementary Information

C Rua et al.

Supplementary Information 1 Demographics for the healthy controls (HC) and Covid+ subgroup data. Abbreviations: NC=data not collected, NA=not applicable, SpO2=blood saturation percentage, bpm=beat/breath per minute, GAD=Generalized Anxiety Disorder (Spitzer et al., 2006), PHQ=patient health questionnaire (Kroenke et al., 2001), mRS=modified Rankin Score (Swieten et al., 1988).

	**HC**			**COVID-19**		
**Scanner Site**	**Site-1**			**Site-1**	**Site-2**	**Statistical test (site-1 vs site-2)**
Subgroup	HC1	HC2	HC3	NA		
N	18	24	9	14	17	NA
Age (y.o.)	42±12	66±5	44±9	53±10	59±13	p = 0.18
Gender (male=M, female=F)	18M,0F	13M, 11F	2M, 7F	5M, 9F	13M, 4F	($\chi^2 = 4.03$, p = 0.13)
Period of hospital admission (days)	-	-		21±34	13±9	p = 0.36
Time from admission to 7T MRI scan (days)	-	-		195±33	214±107	p = 0.20
Time from follow-up clinic to 7T MRI scan (days)	-	-		50±21	115±34	p < 0.001
Highest CRP during admission (mg/L)	-	-		157±144	207±132	p = 0.34
Highest D-Dimer during admission (ng/mL)	-	-		1802±3047	8704±18970	p = 0.19
Lowest platelets during admission (10^9/L)	-	-		223±49	179±43	p = 0.018
WHO severity scale (range: 0-10)	-	-		3.7±2.2	4.9±1.3	p = 0.081
Breathlessness score (range: 0-10)	-	-		2.8±2.4	1.1±1.8	p = 0.11
GAD-7 (range: 0-21)	-	-		4.9±6.0 (range: 0-20)	4.4±4.6 (range: 0-16)	p = 0.84
PHQ-9 (range: 0-27)	-	-		7.2±5.7 (range: 0-15)	6.0±4.1 (range: 2-15)	p = 0.57
mRS	-	-		1.28±1.2	NC	NA

Supplementary Information 2: Registration of the individual QSM maps to standard space

In order to map each subject's χ maps to the 0.5mm isotropic ICBM 2009b standardized space for statistical analysis, the first echo of the T_2^*w imaging data was first registered to each subject's T_1w scan with ANTs. A one step registration was performed using a rigid registration at four convergence levels using cross-correlation for the similarity metric. Then, the T_1w images were mapped to standard space in three steps: first and second steps were performed each with four levels, with a mutual information metric, and with rigid and affine transformations, respectively; while the third step was performed with five convergence levels, a cross-correlation metric, and with the symmetric image normalization transformation method.

Because registration of the 3T T_1w data to standard space can be challenging for brainstem regions, an additional registration step was added to the three-step pipeline described above, using a target brainstem mask to limit the registration search area.

For this registration, five convergence levels with a cross-correlation metric and the symmetric image normalization transformation method were used. The χ maps were then mapped to standard space using the registration estimates described above. χ maps were visually inspected in standard space for quality of registration both for the brain and brainstem regions.

Supplementary Information 3: Differences across imaging site were tested on the COVID group. Boxplots split into site (site-1 (green) vs site-2 (orange)) showing differences in the regional average χ obtained from the Brainstem Atlas. FDR-corrected statistics represented on the boxplots. Legend: ns=not significant, SCP Superior Cerebellar Peduncle.

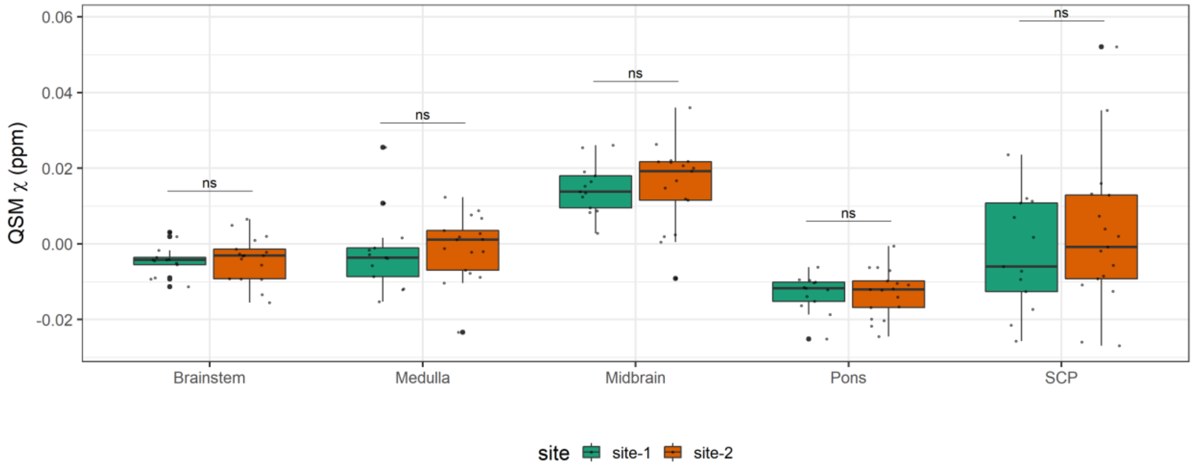

Supplementary Information 4: Some clinical measurements collected from the COVID patients were not available. Therefore, linear mixed effects were performed by dropping patient data that did not include the clinical measurement of interest. Below is a table that describes the linear mixed effect model covariates and the number of subjects available for each test. Abbreviations: PerHospAdm= period of hospital admission, WHO=WHO severity scale, CRP= highest CRP during admission, DD=highest D-Dimer during admission, PLT=lowest platelets during admission, GAD-7= Generalised Anxiety Disorder-7 score, PHQ-9=Patient Health Questionnaire-9, mRS=modified Rankin Scale, TAdmtoScan= time from admission to 7T MRI scan, cluster_index=index of the cluster obtained from the randomise group analysis (subsection 2.4, main manuscript).

Linear mixed effects model	Number of subjects(N)
χ ~ PerHospAdm + age + gender + age*gender + TAdmtoScan + cluster_index	31
χ ~ WHO + age + gender + age*gender + TAdmtoScan + cluster_index	31
χ ~ CRP + age + gender + age*gender + TAdmtoScan + cluster_index	28
χ ~ DD + age + gender + age*gender + TAdmtoScan + cluster_index	25
χ ~ PLT + age + gender + age*gender + TAdmtoScan + cluster_index	28
χ ~ Breathlessness + age + gender + age*gender + TAdmtoScan + cluster_index	21
χ ~ GAD-7 + age + gender + age*gender + TAdmtoScan + cluster_index	24
χ ~ PHQ-9 + age + gender + age*gender + TAdmtoScan + cluster_index	24
χ ~ mRS + age + gender + age*gender + TAdmtoScan + cluster_index	14

Supplementary Information 5: Scatter plots of the average QSM χ obtained on the clusters from the voxelwise group analysis with the period of hospital admission, (A) including all subjects, (B) excluding outlier – subject with period hospital admission = 134 days. The R^2 is also displayed in each plot.

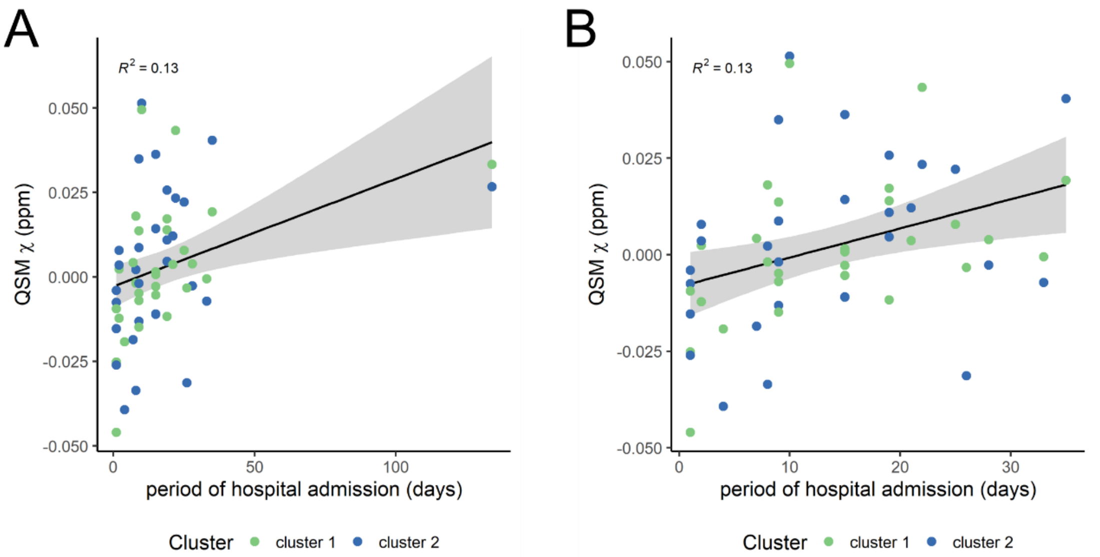

Supplementary Information 6: (A) QSM (B) phase and (C) magnitude data from COVID patient showing lack of viable MR signal in the brainstem. Range of QSM map -0.1 to 0.1 ppm. Range of phase map -3.14 to 3.14 radians, range of magnitude map 0 to 702.

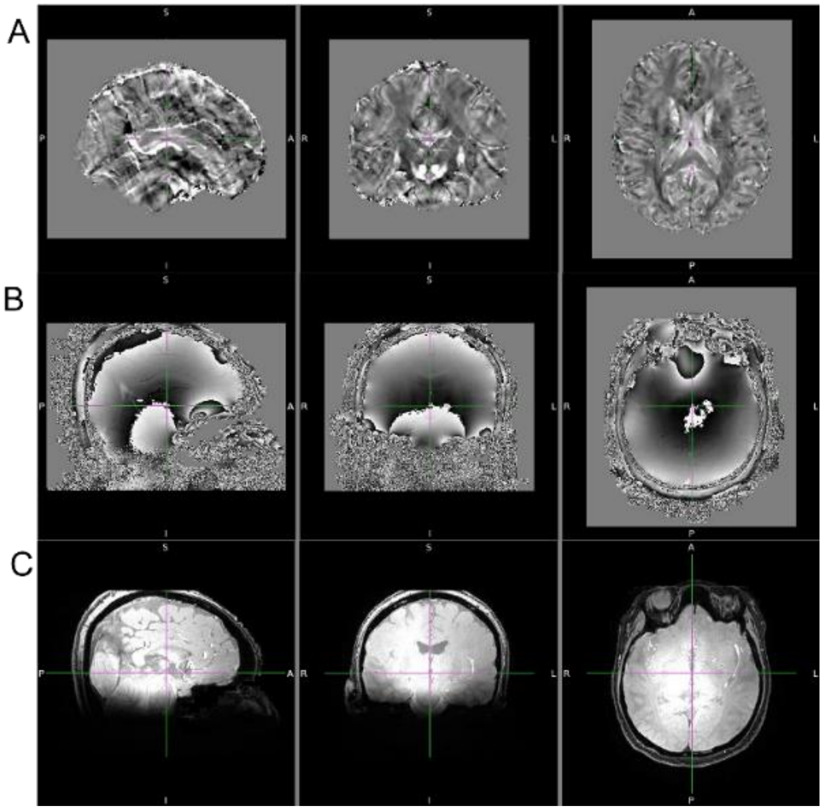

Supplementary Information 7: Cluster regions showing significant increase in QSM χ in the COVID group compared to the healthy control group (TFCE corrected p<0.05, cluster threshold t=2.5). Legend: COG=center of gravity. Brainstem Navigator ROIs that overlapped the significant clusters described in the last two columns.

Cluster #	Volume of cluster [mm^3]	Max t-statistic in Cluster	COG X	COG Y	COG Z	p (FDR)	Cohen's d	95% CI [ppm]	Pr(posterior)	BF	Location	Brainstem Navigator overlapping ROIs	Brainstem Navigator ROI full description
C1	390.25	5.83	182	168	34.5	<0.0001	5.0	[0.0097 0.023]	1.00	2556	Medulla	iMRt_l, iMRt_r, iMRtl_l, iMRtl_r, iMRtm_l, iMRtm_r, ION_r, ROb, RPa, sMRt_l, sMRtl_l, Ve_l, VSM_l, VSM_r	inferior medullary reticular formation (left and right), inferior olivary nucleus (right), raphe obscurus, raphe pallidus, superior medullary reticular formation (left), vestibular nuclei complex (left), viscero-sensory-motor nuclei complex (left and right)
C2	297	4.68	180	180	58.4	<0.0001	5.3	[0.010 0.023]	1.00	22863	Pons, Medulla	ION_r, LDTg_CGPn_r, PCRtA_l, PnO_PnC_l, RPa, Ve_r	inferior olivary nucleus (right), laterodorsal tegmental nucleus – central gray of the rhomboencephalon, paravicellular reticular nucleus - alpha part (left), pontis oralis and caudalis (left), raphe pallidus, vestibular nuclei complex (right)
C3	105.5	4.58	191	219	75.7	<0.0001	5.6	[0.022 0.047]	1.00	7108	Pons	-	
C4	102.5	4.9	203	206	94	<0.0001	4.9	[0.012 0.029]	1.00	3632	Pons, Midbrain	-	
C5	48.875	4.42	189	193	80.3	<0.0001	4.4	[0.0083 0.022]	1.00	773	Pons	-	
C6	20.875	5.21	188	188	39.3	<0.0001	4.7	[0.013 0.033]	1.00	2069	Medulla	-	
C7	16.5	3.85	183	211	59.9	<0.0001	3.7	[0.014 0.047]	0.98	61	Pons	-	
C8	14.75	4.38	162	191	92.2	<0.0001	4.2	[0.0095 0.027]	1.00	349	Pons	-	
C9	10.625	4.29	215	199	79.6	0.0017	3.2	[0.0083 0.036]	0.97	34	Pons	-	
C10	7.75	4.08	157	222	103	0.00027	3.9	[0.018 0.056]	0.99	148	Midbrain	-	
C11	4	3.84	170	222	79.9	0.0022	3.3	[0.014 0.058]	0.95	20	Pons	-	
C12	3.625	4.84	174	222	123	<0.0001	5.0	[0.022 0.051]	1.00	7271	Midbrain	VTA_PBP_r	ventral tegmental area-parabrachial pigmented nucleus complex (right)
C13	2.5	4.79	173	190	136	<0.0001	4.3	[0.017 0.046]	1.00	997	Midbrain	SC_r	superior colliculus (right)
C14	2	3.99	167	159	17.3	0.00020	4.1	[0.010 0.029]	1.00	209	Medulla	-	
C15	1.625	3.38	173	216	72.2	0.0023	3.1	[0.0089 0.041]	0.96	23	Pons	-	
C16	1.625	3.23	201	220	91.4	0.0045	3.1	[0.0095 0.044]	0.91	10	Pons	-	
C17	1.5	3.92	162	227	107	0.000039	4.6	[0.020 0.050]	1.00	1753	Midbrain	PAG	periaqueductal gray
C18	1.5	4.16	178	193	134	0.00043	3.6	[0.017 0.058]	0.99	106	Midbrain	-	
C19	1	3.96	170	209	52.6	0.00043	3.9	[0.014 0.042]	0.99	90	Pons	-	

Supplementary Information 8: 3D projection on the brainstem ROI of the voxelwise analysis showing increased QSM χ on the COVID group compared to healthy controls. Significant clusters determined with randomise function in FSL (TFCE corrected p < 0.05, cluster inference t = 2.5, cluster volume > 1 mm³).

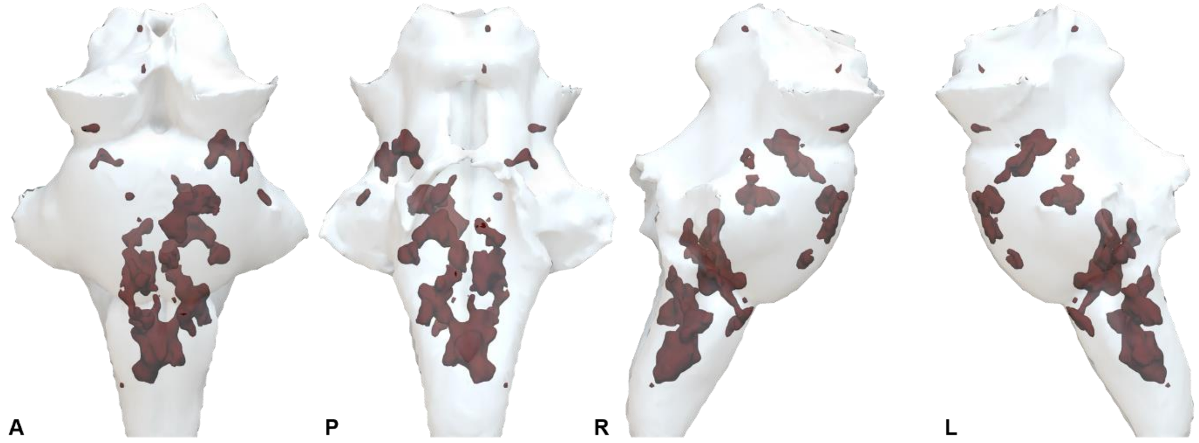

Supplementary Information 9: Overlay of t-statistic scores of the brainstem COVID-vs-HC group voxelwise analysis on the MNI 2009b brain. The results for the FWE corrected p-value < 0.01 (top row) and p-value < 0.05 (bottom row) are displayed for reference.

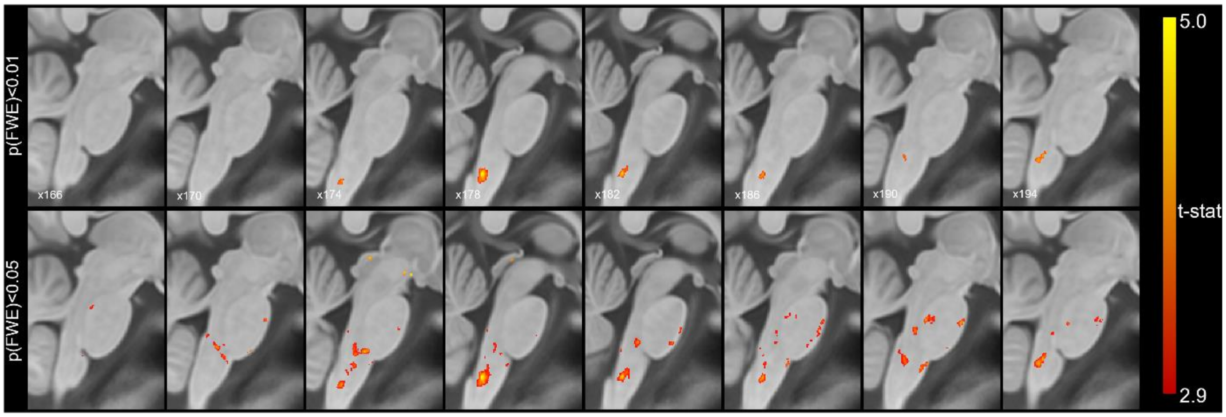

Supplementary Information 10: Table displaying the correlation coefficient (R), p-value, p-posterior and Bayes factor (BF) for the correlation statistics of the QSM χ averaged in the brainstem clusters and the clinical and laboratory outcomes. Abbreviations of fixed effect variables: PerHospAdm= period of hospital admission, WHO=WHO severity scale, CRP= highest CRP during admission, DD=highest D-Dimer during admission, PLT=lowest platelets during admission, GAD-7= Generalised Anxiety Disorder-7 score, PHQ-9=Patient Health Questionnaire-9, mRS=modified Rankin Scale.

		WHO	PerHospAdm	PerHospAdm without outlier	CRP	DD	GAD-7	PHQ-9	PLT	mRS
**Correlation coefficient, R**	main fixed effect	0.40	0.35	0.37	0.36	0.21	0.15	-0.071	0.11	0.60
	main fixed effect	0.046	0.025	0.054	0.041	0.15	0.65	0.94	0.51	0.0046
**lmer, p-value**	ROI effect	0.90	0.90	0.86	0.77	0.84	0.66	0.66	0.77	0.60
	main fixed effect	0.70	0.86	0.76	0.84	0.47	0.28	0.25	0.34	0.94
**p-posterior**	ROI effect	0.21	0.21	0.22	0.22	0.23	0.23	0.23	0.22	0.29
	main fixed effect	2.3	6.1	3.1	5.13	0.88	0.39	0.34	0.52	16.3
**BF**	ROI effect	0.27	0.27	0.28	0.28	0.29	0.30	0.30	0.28	0.40

*Supplementary Information 11: Boxplots of differences in the regional average χ between the COVID group and the healthy control (HC) group obtained from the Harvard-Oxford Atlas. Group differences assessed with a linear model with age and gender, and age by gender interaction added as explanatory variables of no interest. FDR-corrected statistics represented on the boxplots. Legend: *** p<0.001, ** p<0.01, * p<0.05, ns not significant, CSF=cerebral spinal fluid, GM=grey matter, WM=white matter.*

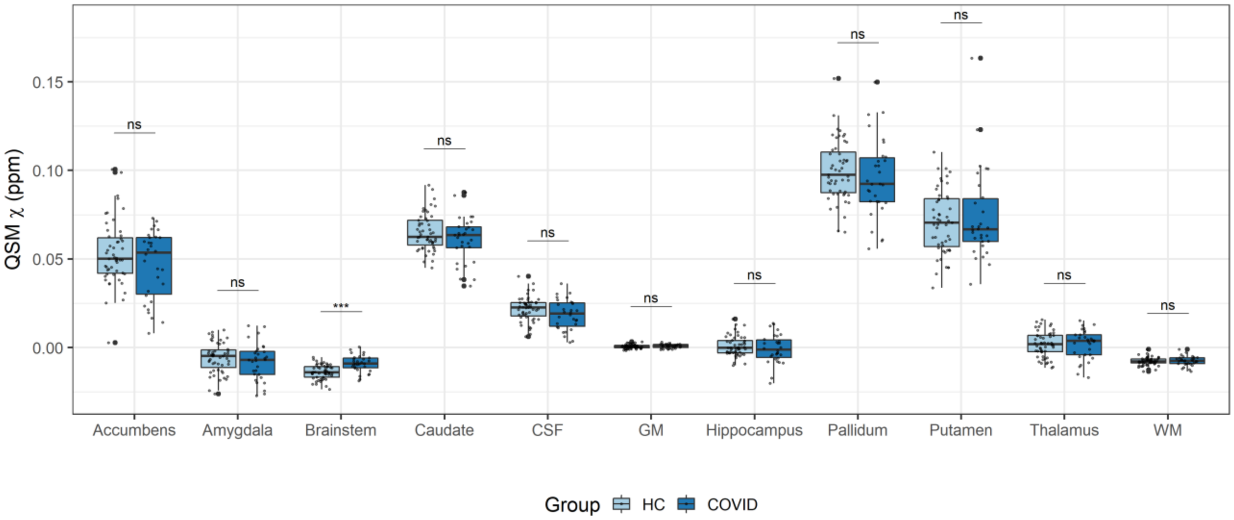

Supplementary Information 12: QSM maps from (A) a healthy control example subject and (B) from the healthy control subject average. The brainstem is outlined in red. Range of QSM map -0.1 to 0.1 ppm.

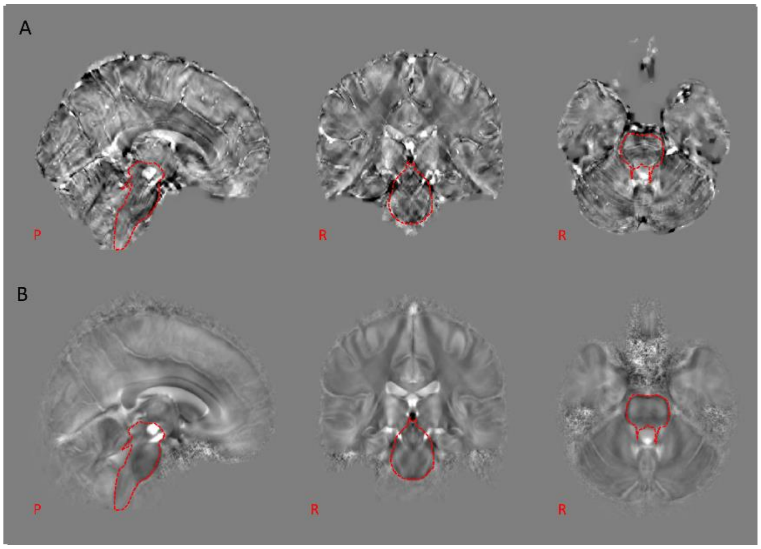

References:

Kroenke, K., Spitzer, R. L., & Williams, J. B. W. (2001). The PHQ-9: Validity of a brief depression severity measure. *Journal of General Internal Medicine*, *16*(9), 606–613. https://doi.org/10.1046/j.1525-1497.2001.016009606.x

Spitzer, R. L., Kroenke, K., Williams, J. B. W., & Löwe, B. (2006). A brief measure for assessing generalized anxiety disorder: The GAD-7. *Archives of Internal Medicine*, *166*(10), 1092–1097. https://doi.org/10.1001/archinte.166.10.1092

Swieten, J. C., Koudstaal, P. J., Visser, M. C., Schouten, H. J. A., & van Gijn, J. (1988). Interobserver agreement for the assessment of handicap in stroke patients. *Stroke*, *19*, 604–607. https://doi.org/10.1161/01.STR.20.6.828
